# Rats and Seabirds: Effects of Egg Size on Predation Risk and the Potential of Conditioned Taste Aversion as a Mitigation Method

**DOI:** 10.1371/journal.pone.0076138

**Published:** 2013-09-18

**Authors:** Lucía Latorre, Asier R. Larrinaga, Luis Santamaría

**Affiliations:** Laboratory of Spatial Ecology, Mediterranean Institute for Advanced Studies (C.S.I.C.-U.I.B.), Esporles, Mallorca, Balearic Islands, Spain; Monash University, Australia

## Abstract

Seabirds nesting on islands are threatened by invasive rodents, such as mice and rats, which may attack eggs, chicks and even adults. The low feasibility of rat eradications on many islands makes the development of alternate control plans necessary. We used a combination of field experiments on a Mediterranean island invaded by black rats ( *Rattus* *rattus* ) to evaluate (1) the predation risk posed to different-sized seabird eggs and (2), the potential of two deterrent methods (electronic and chemical) to reduce its impact. Rats were able to consume eggs of all sizes (12 to 68 g), but survival increased 13 times from the smallest to the largest eggs (which also had more resistant eggshells). Extrapolation to seabird eggs suggests that the smallest species ( *Hydrobates* *pelagicus* ) suffer the most severe predation risk, but even the largest ( *Larus* *michahellis* ) could suffer >60% mortality. Nest attack was not reduced by the deterrents. However, chemical deterrence (conditioned taste aversion by lithium chloride) slowed the increase in predation rate over time, which resulted in a three-fold increase in egg survival to predation as compared to both control and electronic deterrence. At the end of the experimental period, this effect was confirmed by a treatment swap, which showed that conferred protection remains at least 15 days after cessation of the treatment. Results indicate that small seabird species are likely to suffer severe rates of nest predation by rats and that conditioned taste aversion, but not electronic repellents, may represent a suitable method to protect colonies when eradication or control is not feasible or cost-effective.

## Introduction

Biological invasions represent one of the global drivers of biodiversity loss [1,2]. They often alter ecosystem structure and function, and their effects feed back to other elements of global change [3]. Islands are particularly susceptible to species introductions (e.g. 80% of documented bird and mammal introductions took place on islands [4]), which usually cause more intense impacts than on mainland ecosystems ( [5]; but see 6 for plant invaders) owing to the rarity and evolutionary singularity of island biotas (often evolved without natural enemies and therefore lacking defensive traits against them [7,8]). Moreover, because insular biotas tend to be less diverse than continental ones, they offer weaker resistance to biological invasions and greater sensitivity to their effects [9,10], which often include cascades of native-species extinctions [11,12].

Islands are key habitats for nesting seabirds [13], largely because the absence or scarcity of terrestrial predators enhances reproductive success. The consequence of an evolutionary history free of predators at breeding colonies is however, that seabirds often lack defensive mechanisms against them (e.g. inaccessible nests, early independence of chicks), making them more prone to severe impacts from biological invasions by predatory mammals [5,14,15]. Around 60% of the seabird species cited in the IUCN red list are under some level of threat from invasive animals (mainly mammals [16]), most often through their effect on breeding colonies ( [8,17]). Amongst such predators, rats (particularly black rats; *Rattus* *rattus* ) are considered to be a direct cause of the threatened status of at least 75 island-nesting species of seabirds [18].

At present, rats occupy 82% of archipelagos worldwide; most of them outside of their native ranges [14], where they are responsible for the decline and eventual elimination of many native animal species [19-23]. These impacts may arise through direct predation [24], inter-specific competition [25] or indirect effects [26]. In particular, the decline of seabird populations on islands is often triggered by rat invasions [14,18]. In such cases, rat eradication programs (most often based on the application of poisonous bait [27]) are sometimes sufficient to foster the recovery of seabird populations [28-30]. Eradications are starting to succeed on large islands (e.g. Campbell Island or Pinzón Island [30,31], respectively). However, they may be controversial [32] owing to their high economic costs, the environmental risks involved [33] and the potential for collateral damages on non-target species [31,34]. Moreover, they may be followed by re-colonization, if controls against the introduction of new individuals are not feasible or enforceable [35]. In such cases, alternative control techniques may include population-control programs (repeated reductions of rat abundance at particularly relevant or sensitive sites [33,35,36]) or measures aimed at mitigating the rats’ most relevant impacts. Given that such measures are specifically tailored to minimize the impacts of rat presence, a detailed understanding of such impacts is a pre-requisite for their design and cost-effective application. Unfortunately, knowledge of the processes underlying the harmful impacts of invasive rats on island-nesting seabirds is still fairly limited [32,35,37].

During recent decades, several authors have searched for the specific characteristics that make certain seabird species more sensitive to rat predation. Atkinson [14] and Imber [38] indicated that species with burrow or cavity nesting, as well as those included in the *Hydrobatidae* and *Alcidae*, were amongst the most affected. This hypothesis has been confirmed by a recent review [18] but the mechanism by which these species are particularly affected by rats, as compared to ground nesting species and/or those belonging to other families, remains unclear. Suggestions include the effect of nesting “microhabitat” (i.e. the birds’ fossorial habits, which facilitate rats’ access to the nests, and nocturnal activity patterns, which decrease nest defense when rats are also active [18]) and/or nesting ecology (lack of active anti-predator defenses in burrow/cavity nesters, as compared to ground nesters [39,40]; in [18]; [41,42]). Another characteristic that has received less attention, but tends to differentiate burrow/cavity nesters from ground nesters, is their smaller size [18]. Smaller seabirds have been shown to be more sensitive to rat invasions [37], and a review by Jones *et al*.[18]. acknowledges that, owing to the confounding effects of size, family and nesting strategy, the relative effects of these factors remains unresolved.

Nest predation by rats may affect seabird eggs, chicks and adults. Eggs represent a particularly sensitive stage (since they fully depend on incubating adults for protection, apart from their intrinsic protection features such as shell resistance or egg size). Because large eggs are more difficult to manipulate and tend to have thicker eggshells, it has been suggested that egg size may confer resistance against rat predation [14]. Evidence to date is scarce and controversial. On the one hand, Jones et al. [18] could not confirm this hypothesis, although the available data were too scarce for a robust conclusion. On the other hand, field and laboratory experiments with hen eggs offered to black rats [43], Japanese quail and zebra finch eggs offered to white-footed mice [44] and chipmunks [45] and Japanese quail and clay eggs offered to white-footed mice and chipmunks [46] suggest that jaw-gape limitations and/or strong eggshells may constrain the ability of small rodents to depredate on larger eggs, although behavioral naivety may also contribute to the observed responses [43,45].

We tested this hypothesis by means of a field experiment, in which artificial nests containing eggs of four different sizes were subjected to predation by black rats at Sa Dragonera Islet (Mallorca Island), which hosts breeding colonies of several seabird species of contrasting body and egg sizes. In addition, we assessed the effectiveness of two non-invasive methods applied on artificial nests, aimed at reducing egg predation in seabird colonies: (1) Induction of egg deterrence by conditioned taste aversion (CTA [47,48]), using an emetic substance (lithium chloride, LiCl) with persistent aversive effects on rodents (as demonstrated in laboratory and field settings [49,50]), (2) Electronic deterrence by means of commercially-available, ultrasonic rodent repellent [51,52] primarily designed for domestic use and still needing a thorough testing in field conditions ( [53] but see 54). We first assessed whether egg size limits predation by black rats in easily accessible, undefended nests placed at ground level, and if so, examined the functional relationship between egg size and estimated predation risk (in the absence of parental protection). For this purpose, we used commercially available hen and quail eggs that reproduced the range of egg sizes laid by the five species of seabirds present in the study area (Table 1). In a second step, we used artificial nests with the most depredated egg-size category to test whether of the two artificial-deterrence methods provided an effective protection against rat predation. Because egg laying and incubation in seabird colonies may last for several weeks owing to asynchronous laying, we evaluated the effect of both methods for a period of five weeks (see Methods, section 2.3) and included an assessment of the persistence for the most effective method (egg deterrence by CTA) for an additional period of 17 days.

**Table 1:** Morphological features of hen and quail eggs used for the experiments, and seabirds that nest on Sa Dragonera Islet [72].

	Weight (g)		Length (mm)		Width (mm)		Shell Resistance (kg)	
	N	Mean ± s.e.	N	Mean ± s.e.	N	Mean ± s.e.	N	Mean ± s.e.
Quail	12	12.33±0.30	12	34.75±0.13	12	26.28±0.10	16	1.33±1.21
Hen Small	12	54.00±0.56	12	53.59±0.25	12	40.87±0.20	27	4.05±0.92
Hen M	12	59.75±0.73	12	56.19±0.33	12	42.88±0.26	10	4.34±1.04
Hen L	12	67.83±0.75	12	59,85±0.34	12	45.71±0.26	16	3.65±1.00
Storm Petrel				28		21.2		
European Shag				62.9		38.4		
Balearic Shearwater				61.19		42.71		
Cory’s Shearwater				68.1		45.4		
Audouin’s Gull				62.2		43.3		
Yellow-Legged Gull				69.8		48.2		

Storm Petrel ( *Hydrobates* *pelagicus* ), European Shag ( *Phalacrocorax* *aristotelis* ), Balearic Shearwater ( *Puffinus* *mauretanicus* ), Cory’s Shearwater ( *Larus* *auduinii* ), Audouin’s Gull ( *Calonectris* *diomedea* ), Yellow-Legged Gull ( *Larus* *michahellis* ).

## Methods

### Study Site

**Figure 1:**
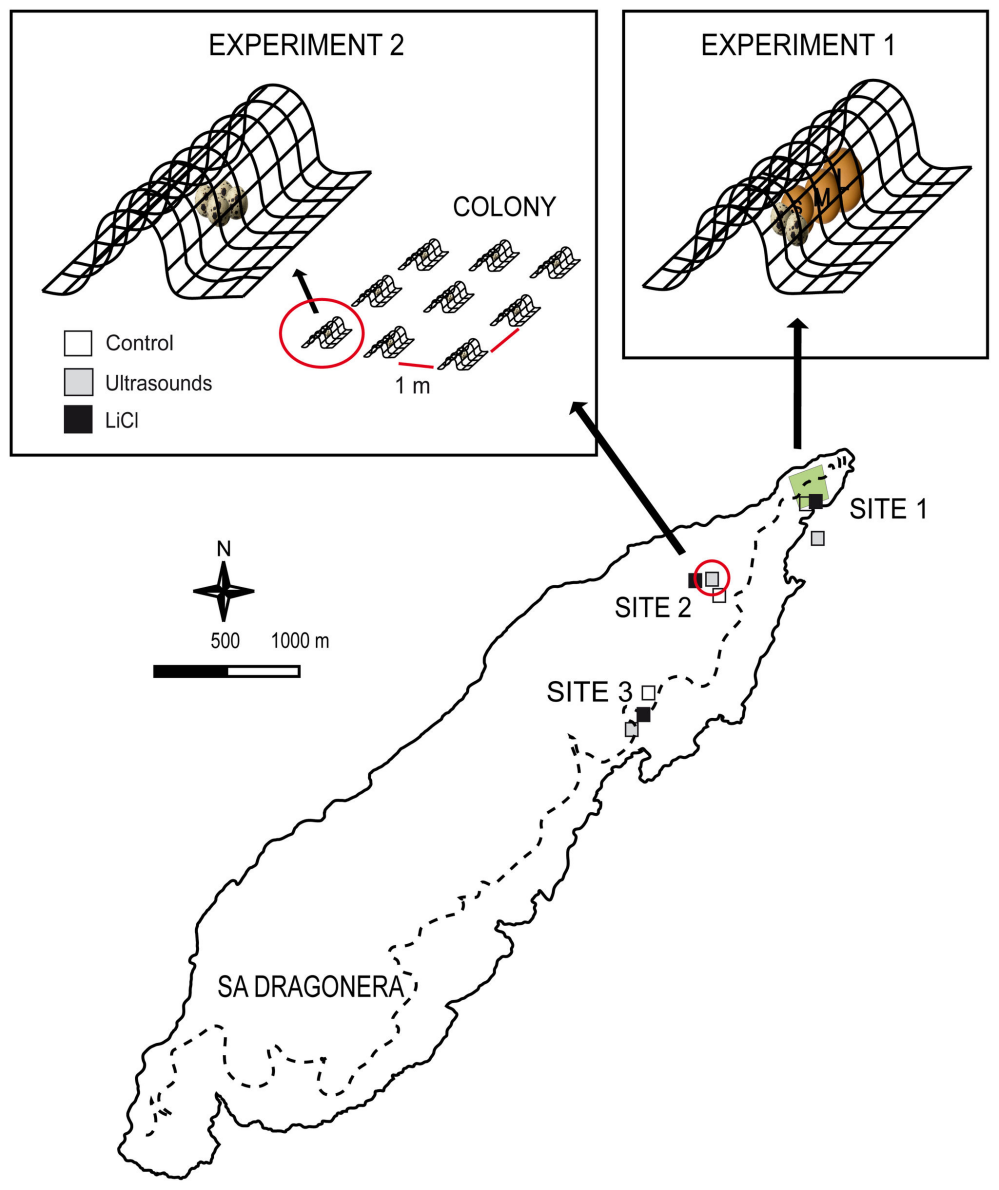
Study area and the three experimental sites in Sa Dragonera Islet. Experiment 1: 30 artificial nests containing different sized eggs were set in an area (large green square) in Site 1. Experiment 2: In sites 1 to 3, three colonies of nine nests (distributed as shown in the left inset) were assigned one of treatments (i.e. one treatment per colony; open squares: control; grey squares: electronic deterrence; black squares: chemical deterrence).

The experiments took place in Sa Dragonera, a small (288 ha) islet located 800 m offshore from Mallorca Island (Balearic Archipelago, 39°35’10.09″ N, 2°19’11.82″; Figure 1). The islet (4 km length and 1 km maximum width) shows a rough topography, with gentle hills facing south and abrupt cliffs at its north face, and a skeletal calcareous substrate. Its climate is semiarid Mediterranean, with low annual rainfall (350 mm) and warm annual mean temperature (17-18 °C [55]). Its vegetation is dominated by sclerophyllous garrigue, with *Pistacia* *lentiscus* *,* *Phyllirea* *angustifolia* and *Olea* *europea* as the most frequent species, interspersed with patches of Aleppo pine ( *Pinus* *halepensis* ) forest and coastal scrub. Due to its central location in the Mediterranean Sea (Figure 1), its rough topography and a history of scarce human presence, Sa Dragonera is an ideal location for nesting seabirds. It was declared as a Special Protected Bird Area in 1979 and, since 1995, has been designated as the marine-terrestrial Natural Park of Sa Dragonera.

Within the north-eastern part of the island, we selected three study sites with comparable topography and vegetation, and contrasting abundances of nesting yellow-legged gulls ( *Larus* *michahellis* , gulls hereafter; Figure 1). Site 1, situated nearby the Tramuntana Cape, had the highest density of gull nests and was situated in the vicinity of a colony of Audouin’s gulls ( *Larus* *audouinii* ). Site 2, located mid-way between Sites 1 and 3, had a low density of gull nests. Site 3, situated nearby the Park’s port and Information Centre, showed an intermediate density of gull nests, and was probably subject to a higher level of anthropogenic influence (although such influence is strongly limited by the Park’s strict regulations).

### Effect of egg size on predation rate

Experiment 1 (effect of egg size on rat predation rate) took place at Site 1. In July 2007, we placed 30 artificial nests containing eggs of four different sizes at randomly-chosen locations across the whole gull breeding colony (minimum distance between nests = 10 m). The experiment started immediately after the gull breeding season (March to June, in Dragonera Islet; González-Mulet J.M. pers. comm.), when rats had already been in contact with natural nests and eggs for several weeks. Half of the nests were placed under shrubs and the other half on open ground without shrub cover. Artificial nests were protected from predation by gulls or other birds by a 50 x 20 cm strip of wire mesh (2 cm mesh-size), fixed to the ground on two sides to form a “tunnel”, which was still accessible to rats.

Each artificial nest contained five eggs: three hen eggs of different commercial sizes (S, M and L) and two quail eggs (we used two instead of one, to reduce differences in profitability between quail and hen eggs). We estimated the egg mass of each type and size class by weighing (to the nearest centigram) a subsample of the experimental eggs (N=12). On a different subsample, we also measured egg length and width (using a digital caliper with 1 mm accuracy), as well as shell resistance (using a Z100 Zwick Universal Materials Testing Machine®). Shell resistance was considered as a surrogate for the difficulty facing a rat that must break the egg shell to be able to consume its content. Egg resistance was defined as the maximum weight (in kg) that the eggs could bear until the first noticeable crack in the shell (Table 1). Once established, artificial nests were observed daily over six days, recording the number of eggs consumed, broken or transported out of each nest; all of these categories were considered as “depredated”.

### Effect of chemical and electronic deterrence on egg predation

Experiment 2 (effectiveness of chemical and electronic deterrents) took place in October and November of 2007, outside of the period of gull nesting, to avoid the confounding effects of natural egg sources. Within each of the three study sites, we set three artificial “colonies” (Figure 1), consisting of nine artificial nests arranged in a 2 x 2 m grid (colony size: 16 m^2^; minimum distance between colonies: 50 m). Each artificial nest contained four eggs of the most-depredated size (quail eggs, see Results, section 3.2) and was protected by a wire-mesh “tunnel” as in experiment 1. The three colonies were located in areas with comparable topography and vegetation, and were randomly assigned to three treatments (one treatment each): a) control, b) chemical deterrence and c) electronic deterrence. Control treatment involved no further manipulation to protect the eggs (i.e. they were freely accessible to black rats). Chemical deterrence consisted of the injection of 0.5 ml of a 3 M solution of LiCl into each egg, calculated to achieve a final concentration 0.15 M within the egg [56]. Electronic deterrence consisted of the installation of a battery-powered “anti-rat” ultrasonic-wave emitter (SC.10RC RADARCAN ®, 20 m^2^ wave range) at the centre of the colony, which broadcast during the entire experiment.

The experiment started at the beginning of October 2007, and continued for four weeks. Every 3 to 5 days (depending on logistic constraints, mainly weather and sea conditions determining access to the islet), we visited the artificial colonies, counted and removed all eggs with any sign of predation (whether consumed, broken or simply moved out of the nest) and replaced them to maintain a constant offering of four eggs per nest throughout the experiment.

To evaluate the persistence of the chemical deterrence effects and obtain a more robust assessment of the relationship between treatments and observed egg-predation rates (given the low number of replicates, N=3), we completed experiment 2 with a treatment shift (which was applied after the 34 days of treatment described above). For this purpose, eggs in the “control colonies” were replaced by eggs treated with LiCl (i.e., they became “control → chemical deterrence” colonies), and those in “chemical deterrence” colonies were replaced by untreated eggs (i.e., they became “chemical deterrence → control” colonies). Due to logistic constraints (poor weather conditions), these colonies could only be visited (and depredated eggs replaced) three times after the treatment shift, with irregular periods between visits (9, 4 and 4 days). In order to compare predation rates measured at equal periods of exposure to predation (four days), we used only the last two post-shift measurements for the analysis, and compared them to the last two measurements in the previous part of the experiment (i.e., before the treatment shift). Hence, the time elapsed from the treatment shift to the first post-shift measurement used in the analysis was 9 days.

In site 1 (Tramuntana Cape), the treatment shift to chemical deterrence was performed in the electronic-deterrence colony instead of the control, because (1) no difference in predation rate was detected in the first part of the experiment between these two treatments, and (2) due to unknown factors, the control colony at that site showed a total absence of predation, which dissuaded us from using it for this final test (see Results, section 3.2).

### Statistical Analysis

The effect of egg size on egg predation (experiment 1) was estimated by fitting Generalized Linear Mixed Models (GLMM; GLIMMIX procedure, SAS v.9.2, SAS Institute 2000) to the final survival after the 6-day period (*survival*). Egg width, weight and breaking resistance were highly inter-correlated; hence, the introduction of more than one of these variables in the same model caused problems of collinearity. To avoid this problem, we fitted separate models with either egg width, weight or breaking resistance as explanatory, continuous response variables. We obtained comparable results for all these models (Table S1) and therefore chose to show solely the results of the one with the best fit - which included egg width as a co-variable. We used the combination of error distribution and link function that provided the best model fit, i.e. a binary distribution and logit link. To ensure the best possible model we tested (by default) several covariance structures of the random effects (autoregressive, unstructured, compound-symmetry, radial smoother, Toeplitz, standard variance…). We combined as well the testing of linear and/or quadratic terms for the continuous factors in the model. We retained the models with the smallest scale term (generalized Chi-square/df).

The effect of chemical and electronic deterrence on egg predation (experiment 2) was analyzed in three steps. First, we analyzed the proportion of nests attacked by rats (i.e. those with at least one depredated egg) within each artificial colony, throughout the initial four-week period, by means of a longitudinal analysis ( [57]; GLIMMIX procedure, SAS v.9; SAS Institute 2000) with a binomial error distribution, a logit link, *treatment* as a fixed, categorical factor and *time* (number of days since the start of the experiment) as a continuous covariate (analogous to a within-subject effect in repeated measures). We fitted a repeated measures model, where the replicate unit was the *colony* (included as a random effect), which was itself nested within *site* (included also as a random effect). We used a random-coefficients model for *time*, which involves a mean slope of change along time (*time* fixed effect) and random contributions of each colony to this common slope (colony*time random effect).

Second, the number of eggs that survived predation within each measurement interval (*survival*) was also modeled by longitudinal analysis, using a linear mixed model (MIXED procedure, SAS® v.9; SAS Institute 2000) with *treatment* as fixed, categorical factor, *time* (as above) as continuous covariate, and random effects for *site* and *colony* (nested within *site*). In this case, and owing to the complexity of temporal effects to be included in the model (see below), we aggregated the data per colony (i.e., we analyzed colony-wise mean survival, instead of survival per individual nest) and subjected it to square-root transformation.

We decided to exclude the data from this control colony at Tramuntana Cape from these analyses. At this specific colony, predation was null throughout the entire experiment. Given the high predation rate in all other control colonies (85.5% of eggs depredated), as well as in the nearest colony (83.5% eggs depredated, under ultrasound treatment), and the close proximity (approx. 50 m distance) to the latter, we find it difficult to understand why rats wouldn’t attack any nests or eggs there. The most parsimonious explanation is non-demoniac intrusion (sensu Hurlbert [58]) - i.e., the interference with an unplanned factor linked to a specific location (such as the presence of a nest or hunting post of nocturnal predators; the near presence of a baiting station used a few months before to protect the Audouin gull colony) or introduced while setting the experiment (although we have not been able to identify any difference in the way we set up this colony compared with the rest). Even if the absence of predation at this specific point is a legitimate effect, indicating that, at some specific locations, rats do no attack eggs, we found it more adequate to exclude it from our specific test of hypothesis for two reasons. First, because including this ‘colony’ made model convergence impossible (owing to zero variance in one of the replicates). Second, and more importantly, because the question that we are addressing in the second experiment was: “in colonies attacked by rats, do electronic and chemical deterrents protect eggs from predation?” Hence, the inclusion of sites without predation provides no scope to test this hypothesis. While the GLMM models for the proportion of nests attacked could be fitted in the absence of these values, in the analysis for mean survival we substituted these data gaps by “neutral” values calculated using Steel and Torrie’s covariate method [59].

Finally, we assessed the effect of the treatment shift using a pre-post analysis based on GLMMs ( [57]; proc GLIMMIX, SAS® v.9, SAS Institute 2000). The number of eggs surviving predation (per nest) was modeled using a Poisson error distribution, a log link function, *period* (pre/post) and *initial treatment* (LiCl or control) as fixed factors, and *site* and *colony* (nested within *site*) as random effects. In addition, the evaluation of the shift “chemical deterrence → control” provided an estimate of the persistence of deterrence effects.

For these three analyses, we ensured the best possible models by combining linear and/or quadratic terms for the continuous factors. We retained the models with the smallest AICc for each case (Table S2, S3 and S4).

### Ethics Statement

The Sa Dragonera Natural Park staff provided permits, accommodation, transportation and technical support during field work. The Scientific-Technical Service of the University of Illes Balears provided infrastructure and technical support for the mechanical tests with eggs. Jocelyn Brito provided advice on the preparation of the chemical deterrent; Martín Piazzón, Rosana Noya and Farid Shami for their help in the field; Maite Louzao, GiacomoTavecchia, Meritxell Genovart, Alejandro Martínez-Abraín and Manuel Igual for sharing their knowledge on seabird biology. L.L. is adhered to the PhD program of the University of Santiago de Compostela. The work reported in this paper did not involve animal experimentation as defined in the Spanish legislation at the moment it was carried out (the "Real Decreto 1201/2005" on the protection of animals used for experimentation and other scientific purposes, available at http://www.boe.es/boe/dias/2005/10/21/pdfs/A34367-34391.pdf). Hence, no animal experimentation protocol and/or permit were required. Field experiments required, however, due permits by the nature conservation authorities, which were requested and granted as needed.

## Results

### Effect of egg size on predation rate

**Figure 2:**
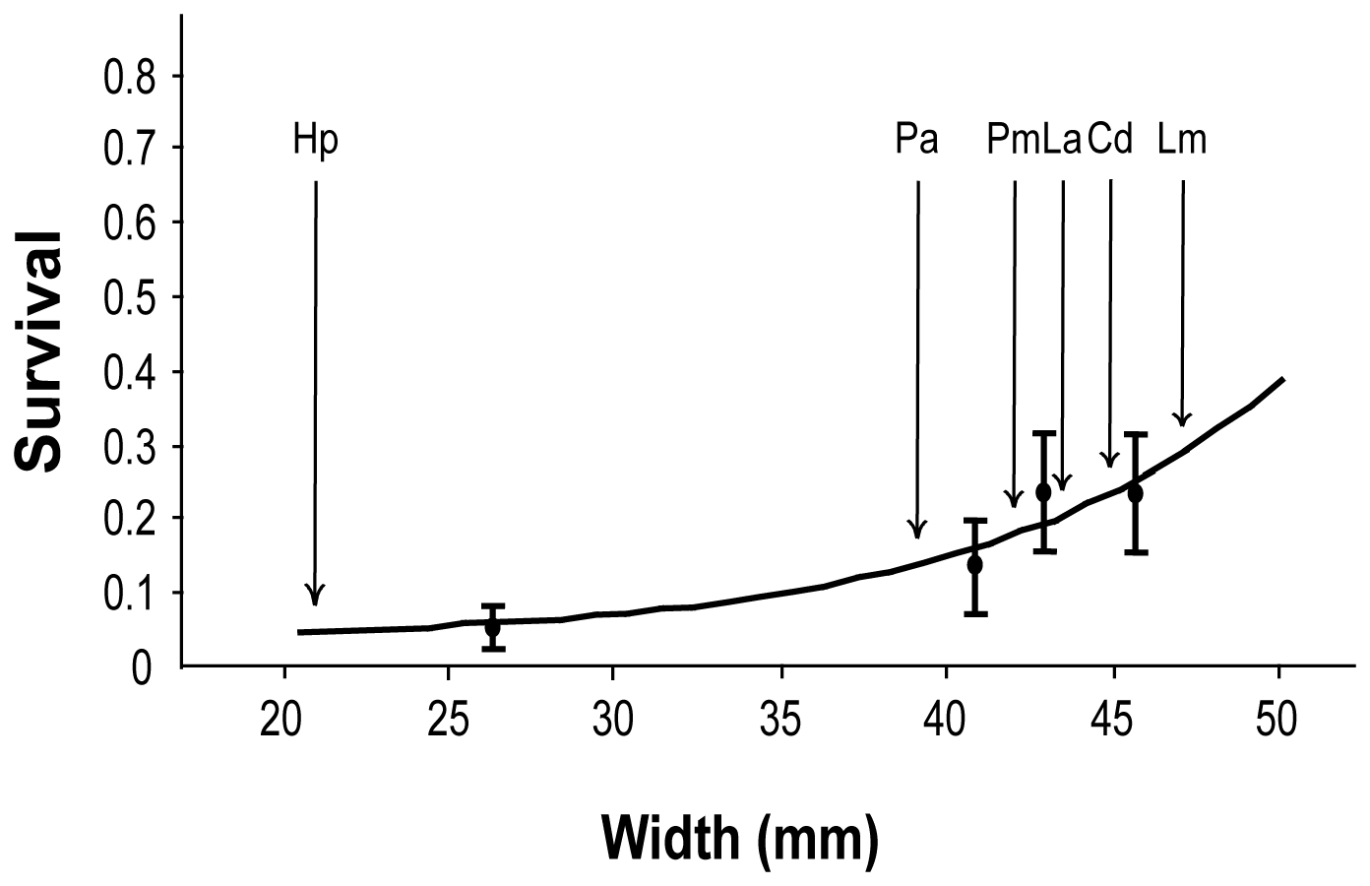
Effect of egg width on survival subjected to predation by black rats ( *Rattus* *rattus* .) Four different sized eggs (quail and S, M and L hen eggs) were offered during six days in undefended nests placed within a breeding colony of yellow-legged gulls. Arrows indicate the estimated survival probabilities of the eggs of six seabird species which nest in Sa Dragonera Islet (Hp: *Hydrobates* *pelagicus* ; Pa: *Phalacrocorax* *aristotelis* ; Pm: *Puffinus* *mauretanicus* ; La: *Larus* *auduinii* ; Cd: *Calonectris* *diomedea* ; Lm: *Larus* *Michahellis*). Lines represent the fits of General Linear Mixed Models.

The majority of eggs (86%) were depredated during the 6-days period, i.e. rats were able to handle, break and consume even the largest hen eggs (45.71 ± 0.26 mm width, Table 1). However, egg survival increased with egg size (F_1,119_ = 9.37; P < 0.005), showing a 13-fold increase for L-size eggs relative to quail eggs (Figure 2). In fact, predation took considerably longer for the largest, as compared to the smallest, eggs (4.6 *versus* 2.9 days, on average).

### Effect of chemical and electronic deterrence on egg predation

**Figure 3:**
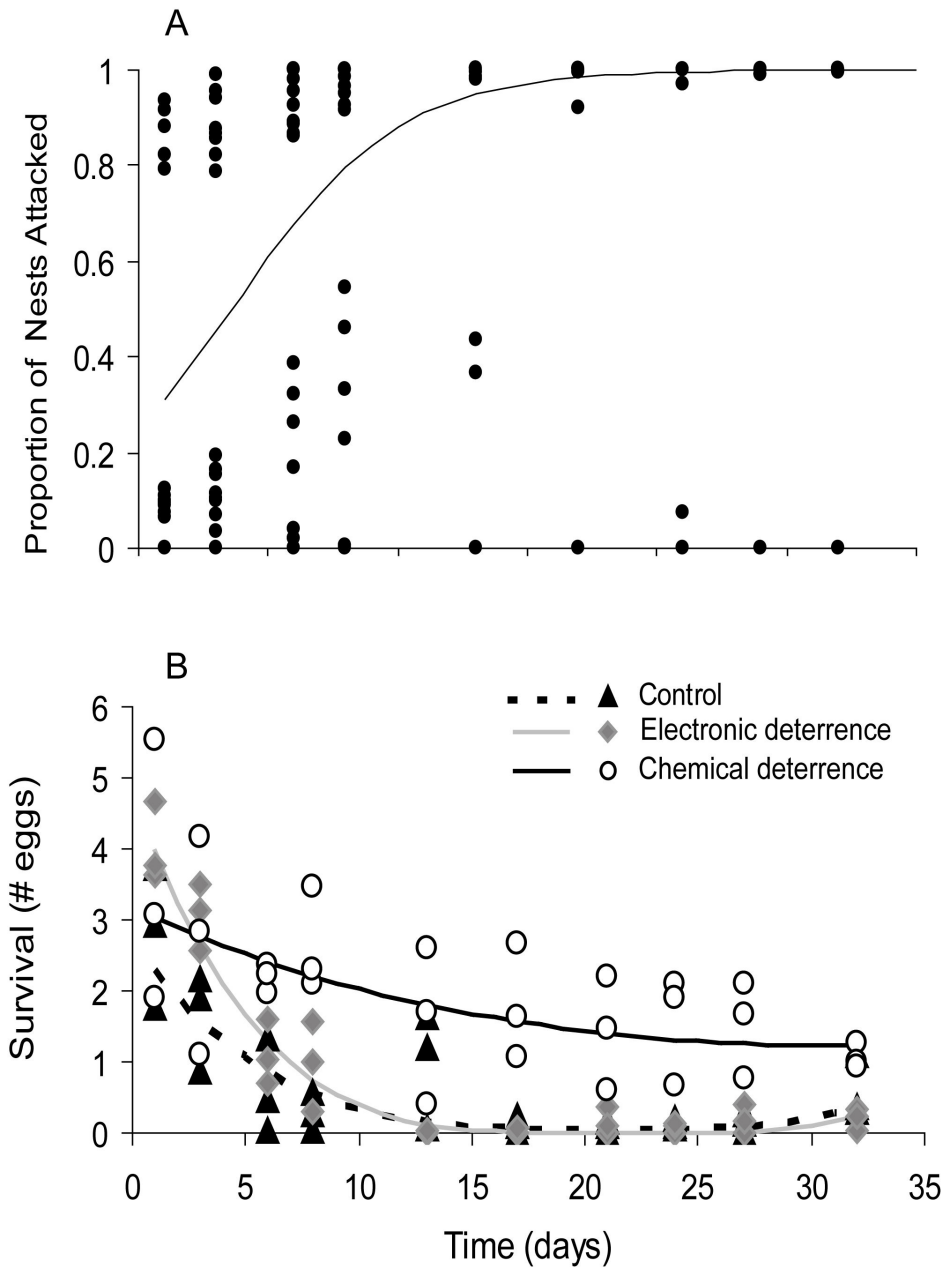
Effect of two deterrence methods on the predation **rates** **of** **quail** **eggs**. Electronic and chemical deterrence were tested for protecting artificial nests simulating seabird colonies. Artificial nests were grouped in “colonies” of nine eggs (see Figure 1). Each data point shows the proportion of nests attacked or eggs consumed since the time at which the previous data point was measured (and all attacked eggs replaced). (A) Nest predation (proportion of attacked nests) was monitored at regular intervals over time (days from the onset of the experiment). Line indicates the effect of time, as estimated from the GLMM model (all treatments pooled) and dots represent partial residuals. (B) The effect of the three treatments (control, electronic deterrence and chemical deterrence) was measured for egg survival (the number of eggs, within each nest, that are still intact at the end of the observation period). Different lines indicate the effect of time for each treatment, as estimated from the GLMM model, and dots represent predicted values.

The proportion of nests that experienced rat predation increased sharply during the first fifteen days of the experiment (from 30 to 90% with all treatments pooled), until reaching an asymptote close to 100% of nests attacked (F_1,4,_ = 6.36; P = 0.652; Figure 3A). The application of chemical and electronic deterrents did not reduce the proportion of nests depredated (treatment: F_2,6_ = 1.65; P > 0.05; days*treatment interaction not included in the final model; Figure 3B).

Conversely, deterrence treatments reduced egg predation throughout the four weeks of the experiment (day*treatment effect, F_2,71_ = 10.17; P < 0.001; *day* ^2^ *treatment effect, F_2,71_ = 7.09; P = 0.002). This was particularly true for the chemical deterrence treatment: the decrease in egg survival along time was less pronounced under this treatment than in control nests (t = -2.8; d.f. = 71; P=0.007 for the linear coefficient and t = 2.52; d.f. = 71; P = 0.014 for the quadratic one) and electronic deterrence (t = -4.46; d.f. = 71; P < 0.001 for the linear coefficient and t= 3.68; d.f.= 71; P < 0.001 for the quadratic one) treatments. As a result, at the end of this phase of the experiment (day 30), colonies protected with chemical deterrence showed less egg predation than those with electronic deterrence (*survival*: mean difference= 1.01, t = 2.80; d.f.= 4; P = 0.049) and marginally less than the controls (*survival*: mean difference= 1.03, t = 2.36; d.f. = 4; P = 0.078). In contrast, electronic deterrence did not increase egg survival to rat predation, neither regarding its temporal pattern (t = 1.67; d.f. = 71; P = 0.0997 for the linear coefficient and t = -1.17; d.f. = 71; P = 0.2479 for the quadratic coefficient) nor its final values (mean difference with control treatment= 0.09, t = 0.44; d.f. = 4; P = 0.682).

The causal relationship between chemical deterrence and increased egg survival was confirmed by the treatment shift (significant period*initial treatment interaction; F_1,205_ = 21.58; P < 0.0001), which showed a larger influence on the “control → deterrence” than on the “deterrence → control” treatment. Hence, following one month of chemical deterrence treatment, suspension of the treatment (i.e. a change to offering unmanipulated, control eggs) did not result in increased predation (F_1,205_ = 0.01; P > 0.05) for at least 15 days (from the treatment shift until the last sampling). In contrast, colonies in the control treatment showed a significant decrease in egg predation after treatment shift (i.e. after being subjected to chemical deterrence; F_1,205_ = 27.29; P < 0.0001).

## Discussion

In our study system, eggs from all sizes tested were attacked and consumed by rats (i.e., we did not detect an upper-size threshold allowing eggs to escape predation, as posed by Atkinson [14], owing to jaw-gap constraints, as suggested by Prieto et al. [43]). However, predation rate decreased with increasing egg size: larger eggs (which are more difficult to bite, handle and break, owing to their bulky dimensions, larger weight and more resistant shell) took longer to be depredated and therefore had higher survival at the end of our observation period (six days). If we assume a positive relationship between eggshell resistance (measured in this paper and positively correlated with egg survival) and eggshell thickness, our results would contrast with those obtained by Jones et al. [18], which reported no significant relation between eggshell thickness and rat predation.

The positive effect of decreasing egg size and resistance on rat-predation risk suggests that, among ground and burrow/cavity nesting seabirds, those with smaller and soft-shelled eggs will suffer larger egg-predation rates when facing rat invasions. Although our results are not directly applicable to real-world nests defended by adults, they indicate that whenever nests are left unattended, smaller and softer eggs will be more susceptible to predation (including both direct consumption at the nest and removal). This effect may be compounded by the smaller body size of the reproductive adults since smaller seabirds, which tend to produce smaller eggs, also show a reduced capacity to defend their clutches against rat attacks. This combination of effects may contribute to the higher sensitivity of smaller seabird species to rat invasions [15,18,37].

In a considerable number of cases, eggs were not consumed directly in the artificial nests, but dragged out of them and consumed outside, often in open ground. This behavior probably reflects a minimization of the risk arising from the potential return of breeding seabird adult, i.e. a potential response of rats to nest defense by adults that may arise from their experience with nesting gulls at the study area.

If we assume that the observed predation rates represent a reasonable, though perhaps conservative surrogate of predation risk, we may conclude that invasions by the black rat are most likely to compromise the breeding success of the smallest seabird present in our study area – the storm petrel ( *Hydrobates* *pelagicus* ) in agreement with Ruffino et al. [15] who found evidence of rat impacts on the populations of this species only. Other endangered seabird species on the island are likely to suffer considerable predation risk (from 0.29 egg^-1^ day^-1^ for Balearic shearwater to 0.28 egg^-1^ day^-1^ for Adouin’s gull) whenever the adults leave the nest unattended. Our results therefore suggest that seabird egg (or body) size should be considered in the evaluation of the potential cost-benefits of rat eradication [60,61]. However, caution is appropriate since numerous factors may condition both the effects of rat presence on nest predation (e.g., nest accessibility, nest defense by adults, rat abundance, availability of alternative food resources) and the likelihood that such effects would translate into changes in population dynamics [62].

As for the potential measures to mitigate egg predation by rats, only chemical deterrence resulted in a significant increase in egg survival, while electronic deterrence showed only a transient effect during the first week of the experiment (Figure 3B). Because egg predation increased over time (Figure 3A), as rats learn and get used to exploiting this new resource (which can be also expected in seabird nesting colonies, where eggs are available within fairly restricted areas for several weeks), the net effect of chemical deterrence was to slow down the buildup of higher predation rates. This effect was non-linear, so that differences in egg survival between control and chemical deterrence colonies increased during the first two weeks of the experiment and remained fairly stable thereafter.

The potential use of chemical deterrence (generation of taste aversion using LiCl) in the wild has been previously explored in different settings, with positive results (e.g., to prevent egg predation by raccoons, *Procyon* *lotor* [63]). This is the first direct proof, to our knowledge, of its effectiveness as a method for controlling seabird egg predation by rats – a necessary step given the species-specificity of the method [64]. While reptiles and raccoons have been shown to have long-lasting aversive responses (up to 7 months [63,64]; respectively), conditioning on rats was only proven in the laboratory and over fairly short periods (up to 3 days [56,65,66]). Our data show, however, that after several weeks of conditioning, taste aversion provided protection to artificial colonies for at least two additional weeks. The use of commercial eggs injected with LiCl therefore provides a potential method for protecting seabird colonies - e.g., by interspersing artificial nests among the actual nests of the colony before or during the nesting period. Further research would be necessary to evaluate, in practice, which specific settings may better serve the purpose of protecting seabird colonies against rat predation. For example, we would need to investigate whether interspersing within the breeding colony artificial nests with chemical deterrents should be done during the breeding period or before it starts. While the latter would have the advantage of creating aversion responses without disturbing the breeding pairs, it would also require a long-lasting persistence of such responses, which may be difficult to achieve under field conditions.

The results obtained in our experiment suggest a low suitability of electronic deterrence to mitigate egg predation in the wild. Our experiment was conducted with commercially-available devices designed primarily for indoor use. The exposure of devices to outdoor conditions could have caused any equipment or battery malfunctioning on ultrasound volume or frequency. This factor is particularly important, as changes in sound frequency may completely alter the potential distress to rats [52,67]. Alternatively, rats may become accustomed to the device’s emissions or learn to tolerate its distress in exchange for a reliable food reward [68]. While we cannot rule out either of these two possibilities, our experiment suggests that the use of available electronic deterrents is probably not effective for rat deterrence in outdoor conditions (see also 69,70 in [71]).

Our results show that larger eggs experience less predation by black rats, although none of the sizes we tested ensured a complete escape from it. The largest size tested, equivalent to that of the largest seabird species present in the study area (Audouin’s and yellow-legged gulls), would still suffer a considerable predation rate (0.28 and 0.21 egg^-1^ day^-1^) if left unattended. The two methods employed to mitigate such predation showed contrasting results: while the use of electronic deterrence only resulted in a limited and brief reduction of egg predation, the use of chemical deterrence showed a considerable reduction in egg predation (e.g., a three-fold increase in egg survival by the end of the experiment) that stabilized over time and persisted for at least two additional weeks after the treatment ended. Our results can be useful for the design of management programs on islands where seabird colonies are affected by rat invasions. Based on these results, we suggest that (1) seabirds laying smaller eggs (and/or having smaller body sizes) should be considered a priority, and (2) that chemical deterrence might be evaluated as a potential alternative to rat control programs whenever eradication is not possible or feasible.

## Supporting Information

Table S1 **Results of the different models fitted for the analysis of the impact of egg traits on depredation by rats.** In the “Model” column, asterisks indicate the model which provided the best goodness of fit. “Scale” refers to the scale parameter (generalized Chi-square/df). Asterisks on F values indicate the level of significance (* p<0.05, ** p<0.01, *** p<0.001, ^NS^ non-significant). All models used a binomial error distribution and a logit link.
(DOC)

Table S2 **Results of the different models fitted for the analysis of the effect of two different artificial deterrents on egg depredation by rats, measured as the proportion of nests attacked in the artificial colonies.** In the “Model” column, asterisks indicate the model which provided the best goodness of fit. “Scale” refers to the scale parameter (generalized Chi-square/df). Asterisks on F values indicate the level of significance (* p<0.05, ** p<0.01, *** p<0.001, ^NS^ non-significant). All models used a binomial error distribution and a logit link.
(DOC)

Table S3 **Results of the different models fitted for the analysis of the effect of two different artificial deterrents on egg depredation by rats, measured as the survival of eggs placed in the artificial colonies.** In the “Model” column, asterisks indicate the model which provided the best goodness of fit. Asterisks on F values indicate the level of significance (* p<0.05, ** p<0.01, *** p<0.001, ^NS^ non-significant).
(DOC)

Table S4 **Results of the different models fitted for the pre-post analysis of a treatment-swap used to test the validity of the effect of two different artificial deterrents on egg depredation by rats, measured as the survival of eggs placed in the artificial colonies.** In the “Model” column, asterisks indicate the model which provided the best goodness of fit. “Scale” refers to the scale parameter (generalized Chi-square/df). The variable *Initial* indicates which treatment was applied first to each experimental unit, while *Days* indicates the number of days from the beginning of each period (i.e., from the onset of each “swap” treatment; see main text for further details). Asterisks on F values indicate the level of significance (* p<0.05, ** p<0.01, *** p<0.001, ^NS^ non-significant). All models used a Poisson error distribution and a log link.
(DOC)
