# Supplementary material for: Rats and Seabirds: Effects of Egg Size on Predation Risk and the Potential of Conditioned Taste Aversion as a Mitigation Method
Source: PLoS One. 2013 Sep 18;8(9):e76138. doi: 10.1371/journal.pone.0076138 (PMC3776756; doi:10.1371/journal.pone.0076138)
Supplement: Table S1 — Results of the different models fitted for the analysis of the impact of egg traits on depredation by rats. In the “Model” column, asterisks indicate the model which provided the best goodness of fit. “Scale” refers to the scale parameter (generalized Chi-square/df). Asterisks on F values indicate the level of significance (* p<0.05, ** p<0.01, *** p<0.001, NS non-significant). All models used a binomial error distribution and a logit link. (DOC) [file pone.0076138.s001.doc]

| Model | Random effects | Effect | D.F. | F | Scale |
| --- | --- | --- | --- | --- | --- |
| 1* | Intercept | Eggshell resistance | 1;119 | 8,25** | 0.29 |
| 2* | Intercept | Egg length | 1;119 | 9,37** | 0.28 |
| 3* | Intercept | Egg width | 1;119 | 9,37** | 0.28 |
