# Supplementary material for: Rats and Seabirds: Effects of Egg Size on Predation Risk and the Potential of Conditioned Taste Aversion as a Mitigation Method
Source: PLoS One. 2013 Sep 18;8(9):e76138. doi: 10.1371/journal.pone.0076138 (PMC3776756; doi:10.1371/journal.pone.0076138)
Supplement: Table S3 — Results of the different models fitted for the analysis of the effect of two different artificial deterrents on egg depredation by rats, measured as the survival of eggs placed in the artificial colonies. In the “Model” column, asterisks indicate the model which provided the best goodness of fit. Asterisks on F values indicate the level of significance (* p<0.05, ** p<0.01, *** p<0.001, NS non-significant). (DOC) [file pone.0076138.s003.doc]

| Model | Random effects | Subject | Effect | D.F. | F | AICc |
| --- | --- | --- | --- | --- | --- | --- |
| 1* | 1.Intercept, Time, Time*Time | Site | Time | 1;2 | 55,17* | 159 |
|  | 2.Intercept | Treatment*site | Time*Time | 1;2 | 43,15* |  |
|  |  |  | Treatment | 2;4 | 1.59 |  |
|  |  |  | Treatmen*Time | 2;71 | 10,17*** |  |
|  |  |  | Treatment*Time*Time | 2;71 | 7,09** |  |
| 2 | 1.Intercept, Time | Site | Time | 1;2 | 11.07 | 165.6 |
|  | 2.Intercept | Treatment*Site | Treatment | 2;4 | 1.8 |  |
|  |  |  | Treatment*Time | 2;76 | 3,75* |  |
