# Supplementary material for: Rats and Seabirds: Effects of Egg Size on Predation Risk and the Potential of Conditioned Taste Aversion as a Mitigation Method
Source: PLoS One. 2013 Sep 18;8(9):e76138. doi: 10.1371/journal.pone.0076138 (PMC3776756; doi:10.1371/journal.pone.0076138)
Supplement: Table S4 — Results of the different models fitted for the pre-post analysis of a treatment-swap used to test the validity of the effect of two different artificial deterrents on egg depredation by rats, measured as the survival of eggs placed in the artificial colonies. In the “Model” column, asterisks indicate the model which provided the best goodness of fit. “Scale” refers to the scale parameter (generalized Chi-square/df). The variable Initial indicates which treatment was applied first to each experimental unit, while Days indicates the number of days from the beginning of each period (i.e., from the onset of each “swap” treatment; see main text for further details). Asterisks on F values indicate the level of significance (* p<0.05, ** p<0.01, *** p<0.001, NS non-significant). All models used a Poisson error distribution and a log link. (DOC) [file pone.0076138.s004.doc]

| Model | Random effects | Subject | Effect | D.F. | F | AICc |
| --- | --- | --- | --- | --- | --- | --- |
| 1* | 1.Intercept | Initial*site | Initial | 1;4 | 0.92 | 491.18 |
|  | 2.Intercept, Days | Site | Period | 1;205 | 14.31*** |  |
|  |  |  | Initial*period | 1;205 | 21.58*** |  |
| 2 | Intercept | Initial*site | Initial | 1;4 | 0.1 | 511.92 |
|  |  |  | Period | 1;207 | 12.74*** |  |
|  |  |  | Initial*period | 1;207 | 8.32** |  |
|  |  |  | Days | 1;207 | 13.53*** |  |
| 3 | 1.Intercept | Initial*Site | Initial | 1;4 | 0.64 | 497.25 |
|  | 2.Intercept, Days, Days*Days | Site | Period | 1;202 | 11.74*** |  |
|  |  |  | Initial*Period | 1;202 | 19.91*** |  |
| 4 | 1.Intercept | Initial*Site | Initial | 1;4 | 0.11 | 511.58 |
|  |  |  | Period | 1;206 | 11.17** |  |
|  |  |  | Initial*Period | 1;206 | 7.95** |  |
|  |  |  | Days | 1;206 | 6.54* |  |
|  |  |  | Days*Days | 1;206 | 2.47 |  |
| 5 | Intercept | Initial*Site | Initial | 1;4 | 0.7 | 492.45 |
|  |  |  | Period | 1;205 | 15.31*** |  |
|  |  |  | Initial*Period | 1;205 | 19.83*** |  |
|  |  |  | Days | 1;2 | 1.45 |  |
| 6 | 1.Intercept | Initial*Site | Initial | 1;4 | 0.68 | 495.08 |
|  | 2.Intercept, Days, Days*Days | Site | Period | 1;202 | 11.58*** |  |
|  |  |  | Initial*Period | 1;202 | 18.89*** |  |
|  |  |  | Days | 1;2 | 6.12 |  |
|  |  |  | Days*Days | 1;2 | 0.68 |  |
